# Medicinal Plant Polyphenols Attenuate Oxidative Stress and Improve Inflammatory and Vasoactive Markers in Cerebral Endothelial Cells during Hyperglycemic Condition

**DOI:** 10.3390/antiox9070573

**Published:** 2020-07-02

**Authors:** Janice Taïlé, Angélique Arcambal, Patricia Clerc, Anne Gauvin-Bialecki, Marie-Paule Gonthier

**Affiliations:** 1 Diabète athérothrombose Thérapies Réunion Océan Indien, INSERM, UMR 1188, Université de La Réunion, 2 rue Maxime Rivière, 97490 Sainte-Clotilde, La Réunion, France; janice.taile@univ-reunion.fr (J.T.); angeliquearcambal@hotmail.fr (A.A.); 2 Laboratoire de Chimie et de Biotechnologie des Produits Naturels, Université de La Réunion, UR 2212, 15 Avenue René Cassin CS 92003, 97744 Saint-Denis CEDEX 9, La Réunion, France; patricia.clerc@univ-reunion.fr (P.C.); anne.bialecki@univ-reunion.fr (A.G.-B.)

**Keywords:** cerebral endothelial cells, hyperglycemia, oxidative stress, inflammation, antioxidant plant polyphenols

## Abstract

Blood-brain barrier endothelial cells are the main targets of diabetes-related hyperglycemia that alters endothelial functions and brain homeostasis. Hyperglycemia-mediated oxidative stress may play a causal role. This study evaluated the protective effects of characterized polyphenol-rich medicinal plant extracts on redox, inflammatory and vasoactive markers on murine bEnd3 cerebral endothelial cells exposed to high glucose concentration. The results show that hyperglycemic condition promoted oxidative stress through increased reactive oxygen species (ROS) levels, deregulated antioxidant superoxide dismutase (SOD) activity, and altered expression of genes encoding Cu/ZnSOD, MnSOD, catalase, glutathione peroxidase (GPx), heme oxygenase-1 (HO-1), NADPH oxidase 4 (Nox4), and nuclear factor erythroid 2-related factor 2 (Nrf2) redox factors. Cell preconditioning with inhibitors of signaling pathways highlights a causal role of nuclear factor kappa B (NFκB), while a protective action of AMP-activated protein kinase (AMPK) on redox changes. The hyperglycemic condition induced a pro-inflammatory response by elevating NFκB gene expression and interleukin-6 (IL-6) secretion, and deregulated the production of endothelin-1 (ET-1), endothelial nitric oxide synthase (eNOS), and nitric oxide (NO) vasoactive markers. Importantly, polyphenolic extracts from *Antirhea borbonica*, *Ayapana triplinervis*, *Dodonaea viscosa*, and *Terminalia bentzoe* French medicinal plants, counteracted high glucose deleterious effects by exhibiting antioxidant and anti-inflammatory properties. In an innovative way, quercetin, caffeic, chlorogenic and gallic acids identified as predominant plant polyphenols, and six related circulating metabolites were found to exert similar benefits. Collectively, these findings demonstrate polyphenol protective action on cerebral endothelial cells during hyperglycemic condition.

## 1. Introduction

The blood-brain barrier (BBB) is composed of microvascular endothelial cells that correspond to a monolayer of flat cells lining the interior surface of blood vessels. These endothelial cells exert important physiological functions in order to maintain the cerebral microenvironment and participate in brain protection [1,2]. More particularly, endothelial cells produce a wide range of factors comprising vasodilators, like nitric oxide (NO) produced by the endothelial nitric oxide synthase (eNOS), and vasoconstrictors, including endothelin-1 (ET-1) [3,4]. Such molecules are essential to regulate the blood pressure and to prevent the formation of aggregates in microvessels which promote stroke [5,6].

During type 2 diabetes, hyperglycemia may alter the endothelial functions, participating to BBB disruption and brain homeostasis loss [7,8,9]. Of note, oxidative stress plays a causal role in the deleterious effects of hyperglycemia on cerebral endothelial cells [6,10,11]. Oxidative stress is defined as an imbalance between the production of reactive oxygen species (ROS) and the rate of their degradation by the endogenous antioxidant defense system, which is composed of various enzymes, such as superoxide dismutase (SOD), catalase, glutathione peroxidase (GPx), and heme oxygenase-1 (HO-1) [12]. ROS overproduction has been associated with several molecular mechanisms, including an alteration of the mitochondrial electron transport chain, the formation of advanced glycation end products, and the activation of specific ROS-producing enzymes such as NADPH oxidase 4 (Nox4), known as the most abundant endothelial Nox isoform [13,14,15,16]. During hyperglycemia-mediated oxidative stress, high levels of ROS lead to endothelial dysfunctions by damaging DNA, proteins, lipids, and by deregulating NO production and the redox sensitive transcription factor, nuclear factor erythroid 2-related factor 2 (Nrf2), which regulates the expression of genes encoding redox enzymes [10,17,18,19,20,21]. Furthermore, high glucose-induced oxidative stress activates the nuclear translocation of the nuclear factor kappa B (NFκB) transcriptional factor that induces the production of pro-inflammatory cytokines, such as interleukin-6 (IL-6) [22]. Conversely, hyperglycemia-caused redox changes may inhibit AMP-activated protein kinase (AMPK) signaling pathway, which exerts anti-inflammatory effects and acts as a key regulator of the endothelial function through induction of eNOS activity and NO vasodilator production [23,24].

There is still a lack of pharmacological strategies that aim to counteract oxidative stress and inflammation on cerebral endothelial cells during hyperglycemia. Interestingly, polyphenols constitute the most abundant antioxidants provided by the human diet through various fruits, vegetables, and medicinal plants [25,26,27,28]. More than 8000 molecules have been identified and classified in main chemical families, including flavonoids, phenolic acids, stilbenes, and lignans [27]. Polyphenols are characterized by the presence of one or several phenolic rings that contribute to their free radical-scavenging and reducing capacities [29,30] and their ability to improve oxidative stress-caused endothelial dysfunction during metabolic disorders, such as type 2 diabetes [31]. Noteworthy, the biological effects of polyphenols depend on their bioavailability as polyphenols are known to be poorly absorbed and extensively catabolized by the gut microflora. Polyphenol circulating metabolites may consist on microbial metabolites, like derivatives of phenylpropionic, phenylacetic, and benzoic acids, and compounds that are produced by both the microbial and tissular metabolism, such as ferulic acid originating from the catabolism of the phenolic acids chlorogenic and caffeic acids [32,33]. From 2012 to 2017, 22 medicinal plants from Réunion Island have been referred in the French Pharmacopeia. Antidiabetic properties were attributed to eight of them, despite the molecular mechanisms involved needing to be elucidated [34]. These medicinal plants include endemic species from the Indian Ocean area, such as *Antirhea borbonica*, *Dodonaea viscosa*, and *Terminalia bentzoe*, as well as exotic species, like *Ayapana triplinervis*. Even if some authors reported anti-inflammatory and antioxidant activities for plants of the same species and genus [35,36], few data exist regarding their possible benefits to reduce diabetes-related metabolic and vascular complications. Our previous studies demonstrated that polyphenolic extracts from medicinal plants, like *Antirhea borbonica* or *Curcuma longa* turmeric from Réunion Island, exert protective effects on adipose cells exposed to metabolic or bacterial stimuli inducing redox and inflammatory damages that are associated with insulin resistance markers [37,38,39]. Recently, we found that *Antirhea borbonica* polyphenols protect against hyperglycemia-mediated alterations in a mouse stroke model by reducing brain infarct volume and hemorrhagic transformation aggravated by hyperglycemia [40].

The present study aimed to evaluate the protective effects of characterized polyphenol-rich extracts from medicinal plants on redox, inflammatory, and vasoactive markers of cerebral endothelial cells that were exposed to hyperglycemic condition. For the first time, four medicinal plants from Réunion Island were selected in the same study for a comparative analysis of their effects on cerebral endothelial cells, based on their antidiabetic properties referenced in the French Pharmacopeia and their economic interest for innovative pharmacological developments, namely *Antirhea borbonica*, *Ayapana triplinervis*, *Dodonaea viscosa*, and *Terminalia bentzoe.* Furthermore, we determined the impact of pure quercetin, caffeic, chlorogenic, and gallic acids detected as major medicinal plant polyphenols, and six polyphenols related circulating metabolites (ferulic, 3,4-dihydroxyphenylpropionic, 3,4-dihydroxyphenylacetic, 3-hydroxyphenylacetic, 3-hydroxybenzoic, and hippuric acids) on cerebral endothelial cells in hyperglycemic condition. 

## 2. Materials and Methods 

### 2.1. Extraction and Identification of Polyphenols from Medicinal Plant Extracts

*A. borbonica*, *A. triplinervis*, *D. viscosa*, and *T. bentzoe* medicinal plants were collected in Réunion Island (France). The botanical identification associated with voucher number of *A. borbonica* (Rubiaceae, RUN052-F), *A. triplinervis* (Asteraceae, TCN-P061), *D. viscosa* (Sapindaceae, TCN-P028), and *T. bentzoe* (Combretaceae, TCN-P009) plants was performed at the University of Réunion Island. After airflow drying at 45 °C, leaves were reduced to powder. A dissolution of each plant powder (2 g) in 10 mL of an aqueous acetonic solution (70%, *v*/*v*, Sigma–Aldrich, St-Louis, MO, USA) was achieved. Subsequently, the mixture was incubated at 4 °C for 90 min, centrifuged at 1400× *g* at 4 °C for 20 min, and the supernatants containing polyphenols were stored at −80 °C until analysis. Ultra-performance liquid chromatography coupled to electrospray ionization-tandem mass spectrometry analysis (UPLC-ESI-MS-MS, Agilent Technologies, Les Ulis, France) was performed to detect phytochemicals, in accordance with the previously published method [38]. The identification of compounds was based on their retention time, and the *m*/*z* ratio of their parent ions and fragments in negative or positive mode at 280 nm. The total polyphenol contents were determined by Folin–Ciocalteu assay, as we previously described [41]. Briefly, 25 μL of each plant extract were added to 125 μL of Folin–Ciolcateu’s reagent (Sigma–Aldrich, St-Louis, MO, USA) and 100 μL of sodium carbonate (Sigma–Aldrich, St-Louis, MO, USA) in a 96-well plate. Absorbance was measured at 760 nm (FLUOstar Optima, Bmg Labtech, Cambridge, UK) after an incubation for 5 min. at 54 °C, and then during 5 min. at 4 °C. A standard solution of gallic acid (Sigma–Aldrich, St-Louis, MO, USA) was used for calibration. The total polyphenol contents were expressed as g gallic acid equivalent (GAE)/100 g medicinal plant powder. 

### 2.2. Evaluation of Free Radical-Scavenging and Reducing Capacities of Medicinal Plant Extracts

The 2,2-diphenyl-1-picrylhydrazyl (DPPH) assay was performed to assess the ability of polyphenol-rich plant extracts to exert free radical-scavenging and reducing capacities, according to the method previously described [41]. Briefly, 40 μL of each plant extract or a pure polyphenol (100 µM), namely quercetin, caffeic, chlorogenic, or gallic acids (Sigma–Aldrich, St-Louis, MO, USA), or vitamin C used as a positive control (100 μM) were placed in a 96-well plate. Subsequently, 200 μL of 0.25 mM DPPH diluted in methanol (Sigma–Aldrich, St-Louis, MO, USA) were added to each well. After an incubation at 25 °C for 25 min., the absorbance was measured at 517 nm (FLUOstar Optima, Bmg Labtech, Cambridge, UK). Reduced DPPH percentage was used in order to calculate the antioxidant capacity, by the following formula:

Antioxidant capacity (%) = [(Absorbance control − Absorbance sample)/Absorbance control] × 100

### 2.3. Endothelial Cell Culture

Murine bEnd3 cerebral endothelial cells were obtained from the American Type Culture Collection (ATCC^®^ CRL-2299™, Manassas, VA, USA) and cultured in Dulbecco’s Modified Eagle Medium (DMEM) containing 25 mM glucose, 10% heat-inactivated fetal bovine serum, 5 mM L-glutamine, 50 µU/mL penicillin, and 2 µg/mL streptomycin (Pan Biotech, Dutscher, Brumath, France). For the bEnd3-Blue cell line model, the bEnd3 cells were transfected with the plasmid pNiFty-secreted alkaline phosphatase (pNiFty2-SEAP, Invivogen, Toulouse, France) using lipofectamine 3000 (ThermoFischer Scientific, Les Ulis, France) and selection with Zeocin™ at 200 μg/mL (Invivogen, Toulouse, France) during three weeks, according to the previously published method [40]. pNiFty2-SEAP plasmid contains five specific NFκB repeated transcription factor binding sites, an endothelial cell-leukocyte adhesion molecule proximal promoter and a SEAP reporter gene. bEnd3-Blue cells were cultured in complete DMEM containing 200 μg/mL of Zeocin™. Cells were maintained in a humidified 5% CO_2_ incubator at 37 °C.

### 2.4. Evaluation of Cell Viability and Mitochondrial Metabolic Activity

The cells were seeded in a 24-well plate (7.5 × 10^4^ cells/well) during 24 h. Subsequently, the medium was removed and cells treated with each plant extract (10 µM GAE), or with a pure polyphenol (quercetin, caffeic, chlorogenic, or gallic acids, 10 µM), or with a related circulating metabolite (ferulic, 3,4-dihydroxyphenylpropionic, 3,4-dihydroxyphenylacetic, 3-hydroxyphenylacetic, 3-hydroxybenzoic, or hippuric acids, 10 µM, Sigma–Aldrich, St-Louis, MO, USA) during 24 h. Each polyphenolic extract or pure compound was diluted in complete DMEM to maintain cells in the culture medium recommended by ATCC. Next, cell culture medium was eliminated, phosphate buffer saline (PBS) was used to wash cells once, and trypsin-EDTA (Pan Biotech, Dutscher, Brumath, France) was added to detach cells. After centrifugation (500× *g*, 4 min., 25 °C), cell staining was performed with Trypan blue solution (Sigma–Aldrich, St-Louis, MO, USA) and cell counting achieved in a Malassez chamber.

The mitochondrial metabolic activity of cells was measured by using the 3-(4,5-dimethyl-thiazol-2-yl)-2,5-diphenyl tetrazolium bromide (MTT) assay, according to the method previously described [41]. Briefly, the cells were seeded in a 96-well plate (1 × 10^4^ cells/well) during 24 h. Subsequently, the medium was removed and cells were treated with each plant extract (10 µM GAE), or with a pure polyphenol (quercetin, caffeic, chlorogenic or gallic acids, 10 µM), or with a related circulating metabolite (ferulic, 3,4-dihydroxyphenylpropionic, 3,4-dihydroxyphenylacetic, 3-hydroxyphenylacetic, 3-hydroxybenzoic, or hippuric acids, 10 µM) for 24 h. Five hours before the end of the experiment, 20 μL of 5 mg/mL MTT reagent (Sigma–Aldrich, St-Louis, MO, USA) diluted in PBS were added in each well. After centrifugation at 500× *g* for 4 min. at 25 °C, the medium was removed and 200 μL of dimethylsulfoxide (DMSO) were added in each well, in order to dissolve formazan crystals originating from MTT mitochondrial metabolism in living cells. Absorbance was measured at 560 nm (TECAN, Männedorf, Switzerland). 

### 2.5. Measurement of Intracellular ROS Levels

The ROS levels were measured using the fluorogenic probe 2’,7’-dichlorodihydrofluorescein diacetate (DCFH-DA) assay, in accordance with the method previously reported [41]. Cells were seeded in a 96-well black plate (1.8 × 10^4^ cells/well) in DMEM containing 5.5 mM glucose (normoglycemic condition) for 24 h. Subsequently, the medium was removed, cells rinsed twice with PBS, and 100 μL of PBS containing 10 μM of DCFH-DA (Sigma–Aldrich, St-Louis, MO, USA) added in each well. After incubation in a humidified atmosphere (5% CO_2_, 37 °C,) for 45 min., PBS containing DCFH-DA was removed and cells were treated with each plant extract (10 µM GAE), or with a pure polyphenol (quercetin, caffeic, chlorogenic, or gallic acids, 10 µM), or with a related circulating metabolite (ferulic, 3,4-dihydroxyphenylpropionic, 3,4-dihydroxyphenylacetic, 3-hydroxyphenylacetic, 3-hydroxybenzoic or hippuric acids, 10 µM) in hyperglycemic condition (33 mM glucose) or in normoglycemic condition (5.5 mM glucose). After 3 h, fluorescence measurement was performed at the excitation and emission wavelengths of 492 nm and 520 nm, respectively (FLUOstar Optima, Bmg Labtech, Cambridge, UK). For ROS level determination in cells that were exposed to specific inhibitors of intracellular signaling pathways, cells were pre-incubated with 10 μM of inhibitors targeting NFκB (JSH-23) or AMPK (Compound C) in a humidified atmosphere (5% of CO_2_, 37 °C) for 1 h. Then, as described above, cells were treated with PBS containing DCFH-DA for 45 min. before exposition to each plant extract or pure polyphenol in hyperglycemic condition for 3 h. Next, fluorescence measurement was conducted at the excitation and emission wavelengths of 492 nm and 520 nm, respectively (FLUOstar Optima, Bmg Labtech, Cambridge, UK). 

### 2.6. Determination of Antioxidant SOD Activity

The cells were seeded in a six-well plate (3.5 × 10^5^ cell/well) in DMEM containing 5.5 mM glucose (normoglycemic condition) during 24 h. Subsequently, the medium was removed and cells were treated with each plant extract (10 µM GAE) or a pure polyphenol, namely caffeic acid, chlorogenic acid, gallic acid, or quercetin (10 μM) in hyperglycemic condition (33 mM glucose), or in normoglycemic condition (5.5 mM glucose) for 3 h. Next, the medium was removed and cells were washed with PBS, scrapped, and pooled in a phosphate buffer containing KH_2_PO_4_ (100 mM) and DTT (1 mM), pH 7.4. Next, a freeze-thaw step consisting on four cycles of fast freezing in liquid nitrogen and subsequent thawing in 37 °C water bath was done in order to lyse cells, as we reported previously [42]. After centrifugation at 500× *g* for 4 min. at 25 °C, supernatants containing proteins were collected. The protein contents were determined using the bicinchoninic acid assay (BCA) [43] and a calibration curve built with standard bovine serum albumin. The SOD activity of protein lysates were measured according to the method that was described by Bitar et al. [44], by calculating the reduction of acetylated cytochrome C by superoxide radicals produced by the xanthine/xanthine oxidase system. Total SOD activity was measured in a reagent buffer at pH 7.8 with xanthine oxidase (1–2 U/mg protein), xanthine (0.5 mM), cytochrome C (0.2 mM), KH_2_PO_4_ (50 mM), and EDTA (2 mM). MnSOD activity was determined similarly, after the addition of NaCN (1 mM) that leads to a specific inhibition of Cu/ZnSOD activity. Enzymatic activities were measured every 5 s during 3 min. at 25 °C, at a wavelength of 550 nm (FLUOstar Optima, BMG Labtech, Cambridge, UK), and they were expressed as U/g protein. 

### 2.7. Measurement of Intracellular NO Levels

The 4-amino-5-methylamino-2’,7’-difluorofluorescein (DAF-FM) diacetate was used to determine intracellular NO levels, according to the method previously described [42]. Briefly, the cells were seeded in a 96-well black plate (1.8 × 10^4^ cells/well) in DMEM containing 5.5 mM glucose (normoglycemic condition) for 24 h. Subsequently, the medium was removed and cells treated with each plant extract (10 µM GAE), or with a pure polyphenol (quercetin, caffeic, chlorogenic, or gallic acids, 10 µM), or with a related circulating metabolite (ferulic, 3,4-dihydroxyphenylpropionic, 3,4-dihydroxyphenylacetic, 3-hydroxyphenylacetic, 3-hydroxybenzoic, or hippuric acids, 10 µM) in hyperglycemic condition (33 mM glucose) or in normoglycemic condition (5.5 mM glucose) for 3 h. Next, the medium was removed, cells were washed once with PBS and 100 µL of PBS containing 5 µM of DAF-FM (Sigma–Aldrich, St-Louis, MO, USA) added in each well. After incubation in a humidified atmosphere (5% CO_2_, 37 °C,) for 45 min., PBS containing DAF-FM was removed, and cells were treated with 100 µL of insulin (1 µg/mL, Sigma Aldrich, St-Louis, MO, USA) for 2 h. Fluorescence measurement was performed at the excitation and emission wavelengths of 492 nm and 520 nm, respectively (FLUOstar Optima, Bmg Labtech, Cambridge, UK).

### 2.8. Evaluation of NFκB/SEAP Activity

Murine bEnd3-Blue cells expressing NFκB/SEAP reporter gene were cultured in a 96-well black plate (1.8 × 10^4^ cells/well) in DMEM containing 5.5 mM of glucose (normoglycemic condition) for 24 h. The day after, the medium was removed and cells were treated for 3 h with a polyphenol related circulating metabolite (ferulic, 3,4-dihydroxyphenylpropionic, 3,4-dihydroxyphenylacetic, 3-hydroxyphenylacetic, 3-hydroxybenzoic or hippuric acids, 10 µM) in hyperglycemic condition (33 mM glucose) or in normoglycemic condition (5.5 mM glucose). NFκB/SEAP activity was evaluated with Quanti-Blue assay (Invivogen, Toulouse, France) and absorbance was measured at 620–655 nm (FLUOstar Omega, Bmg Labtech, Cambridge, UK).

### 2.9. Quantification of Cytokine Secretion 

The cells were seeded in a six-well plate (3.5 × 10^5^ cells/well) in DMEM containing 5.5 mM glucose (normoglycemic condition) for 24 h. Subsequently, the medium was removed and cells treated with each plant extract (10 µM GAE), or with a pure polyphenol (quercetin, caffeic, chlorogenic or gallic acids, 10 µM), or with a related circulating metabolite (ferulic, 3,4-dihydroxyphenylpropionic, 3,4-dihydroxyphenylacetic, 3-hydroxyphenylacetic, 3-hydroxybenzoic or hippuric acids, 10 µM) in hyperglycemic condition (33 mM glucose), or in normoglycemic condition (5.5 mM glucose) for 8 h. Next, cell culture media were collected and analyzed using Mouse IL-6 ELISA kit (eBioscience, ThermoFisher Scientific, Dardilly, France). For cellular protein extraction, 700 μL of PBS were added to each well. After scrapping, cells were collected and centrifuged at 500× *g* for 4 min. at 25 °C. Supernatants were removed and 200 μL of lysis buffer were added (Tris 25mM pH 8.3, KCl 10 mM, DTT 1 mM, EDTA 1 mM, Triton X-100 1%, protease inhibitors 1 X). After resuspension of cell pellet in lysis buffer, a centrifugation at 500× *g* for 4 min. at 25 °C was performed and supernatants containing proteins were collected. Total cellular protein contents were determined by BCA assay and absolute values of IL-6 were normalized to these contents. 

### 2.10. Evaluation of Gene Expression

The cells were seeded in a six-well plate (3.5 × 10^5^ cells/ well) in DMEM containing 5.5 mM of glucose (normoglycemic condition) during 24 h. Subsequently, the medium was removed and cells treated with each plant extract (10 µM GAE) or a pure polyphenol, namely caffeic acid, chlorogenic acid, gallic acid or quercetin (10 μM) in hyperglycemic condition (33 mM glucose), or in normoglycemic condition (5.5 mM glucose) for 6 h. Next, total RNA was isolated with TRIzol™ (Invitrogen, ThermoFisher Scientific, Dardilly, France). Next, 4 µg of RNA were reverse-transcribed (RT) using Random hexamer primers (Eurogentec, Liège, Belgium) with Superscript^TM^ II (Invitrogen, ThermoFisher Scientific, Dardilly, France). The quantitative polymerase chain reaction (qPCR) was achieved by using fast SYBR green^TM^ master Mix (Applied Biosystems, ThermoFisher Scientific, Dardilly, France). The relative expression of genes coding for redox factors (catalase, Cu/ZnSOD, GPx, HO-1, MnSOD, Nox4, and Nrf2), the pro-inflammatory mediator NFκB and vasoactive markers (eNOS, ET-1) was normalized to the expression rate of glyceraldehyde-3-phosphate dehydrogenase (GAPDH) gene. Table 1 reports primer sequences. The results were obtained through analysis by 7500 system SDS software (Applied Biosystems, ThermoFisher Scientific, Dardilly, France). 

### 2.11. Statistical Analysis

Data were expressed as means ± SEM of three independent experiments (three cellular passages). Statistical analysis was conducted through one-way analysis of variance (ANOVA) followed by the Bonferroni’s multiple comparison test, and the Pearson linear correlation was established when appropriate by the program Graph-Pad Prism 6 (GraphPad Software, Inc., San Diego, CA, USA). Statistically significant differences were considered for a *p* value < 0.05.

## 3. Results

### 3.1. Characterization of Polyphenols Extracted from Medicinal Plants and Evaluation of Their Free Radical-Scavenging and Reducing Activities

**Figure 1:**
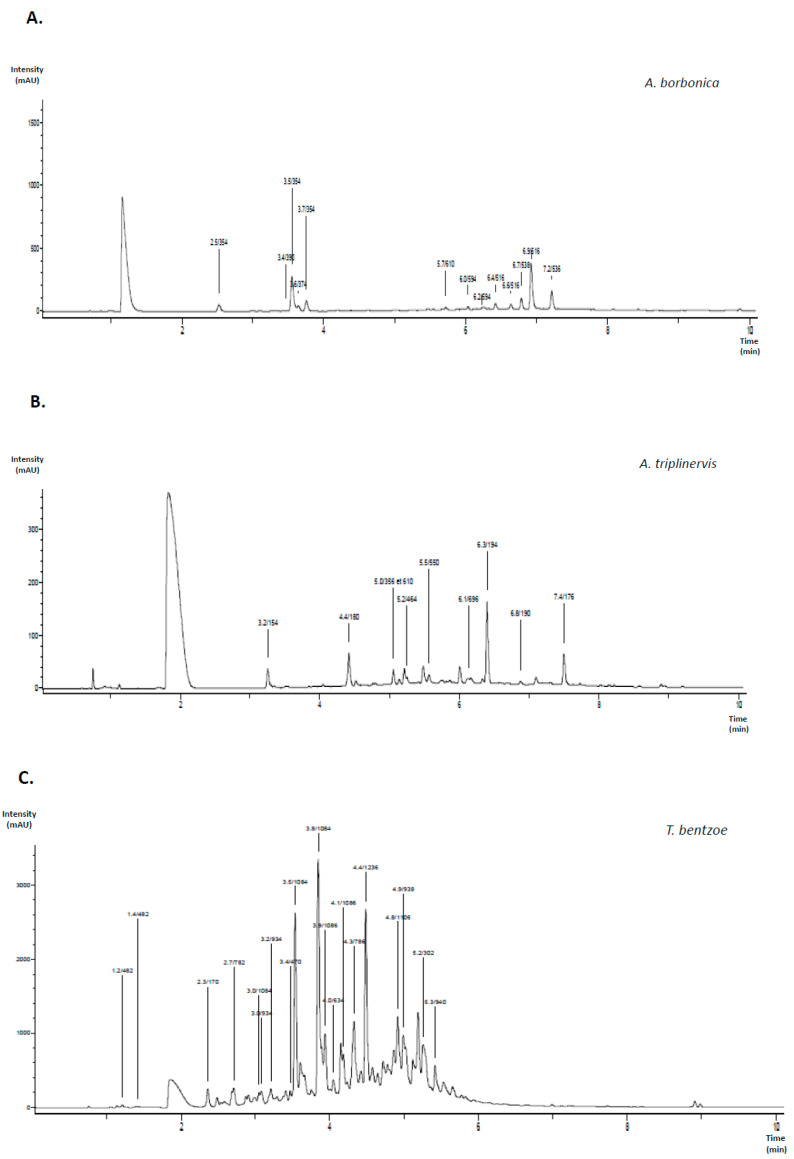

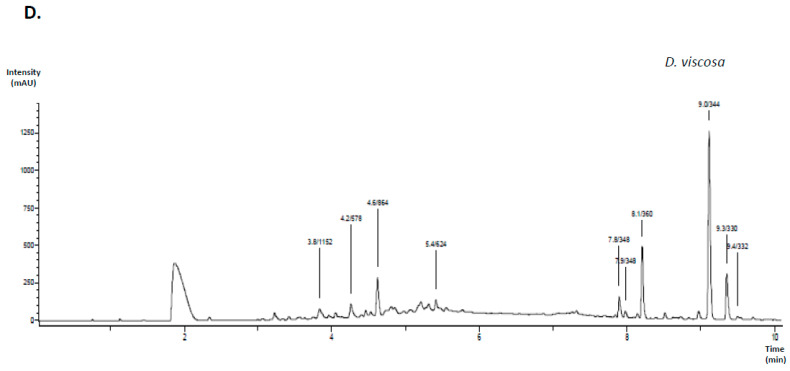
Identification of polyphenols from medicinal plant extracts. Polyphenols extracted from the medicinal plants *Antirhea borbonica* (**A**), *Ayapana triplinervis* (**B**), *Terminalia bentzoe* (**C**), and *Dodonaea viscosa* (**D**) were identified by Ultra-performance liquid chromatography coupled to electrospray ionization-tandem mass spectrometry analysis (UPLC-ESI-MS-MS) at 280 nm.

UPLC-ESI-MS-MS analysis (Figure 1A) shows that polyphenols detected in *A. borbonica* plant extract were mainly phenolic acids, including chlorogenic and dicaffeoylquinic acids, which are derivatives of caffeic acid, and quercetin and kaempferol glycosides from the flavonoid family (Table 2). Polyphenols that were depicted in *A. triplinervis* plant extract were the phenolic acids protocatechuic, isoferulic, and caffeic acids (Figure 1B). Of note, UPLC-ESI-MS-MS analysis also led to identifying both ayapin and ayapanin, known as specific coumarin derivatives in *A. triplinervis* plant. Regarding *T. bentzoe* plant extract, different polyphenols that were derived from gallic and ellagic acids were detected, such as the ellagitannins punicalin and punicalagin (Figure 1C). *D. viscosa* plant extract was characterized by the presence of different flavonoids, including the methylated form of quercetin called isorhamnetin, a trimethoxyflavone and procyanidins (Figure 1D). Total polyphenol contents ranged from 0.5 to 8.1 g GAE/100 g plant, depending on the medicinal plant extract considered, as reported in Table 3. *T. bentzoe* plant was identified as the richest source of polyphenols, followed by both *A. borbonica* and *D. viscosa* plants exhibiting similar ranges of total polyphenol quantities that were 2.5-fold lower than that of *T. bentzoe* plant. In comparison, the total polyphenol content of *A. triplinervis* plant was six- to 14-fold lower than those of other medicinal plants. To evaluate whether the polyphenols present in plant extracts exhibit free-radical scavenging and reducing capacities, DPPH assay was performed using vitamin C as a positive control. Given that UPLC-ESI-MS-MS analysis led to identify caffeic acid in both *A. borbonica* and *A. triplinervis* plants, chlorogenic acid in *A. borbonica* plant, gallic acid in *T. bentzoe* plant, and quercetin in *A. borbonica*, *A. triplinervis*, and *D. viscosa* plants, the corresponding standard polyphenols were also assessed for DPPH assay. The data show that all polyphenol-rich plant extracts were able to scavenge and reduce DPPH radical (Table 3). Polyphenol-rich extracts from *T. bentzoe*, *D. viscosa*, and *A. borbonica* exerted the most pronounced antioxidant activity as compared to that of vitamin C. Interestingly, the standard polyphenols quercetin, caffeic, chlorogenic, and gallic acids exhibited radical-scavenging and reducing capacities, which suggested their possible contribution to the antioxidant properties of the medicinal plants. 

### 3.2. Effect of Polyphenols on the Viability of Cerebral Endothelial Cells 

**Figure 2:**
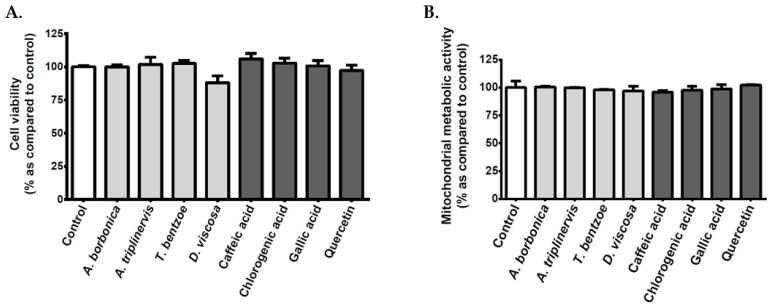
Effect of polyphenols on the viability and mitochondrial metabolic activity of cerebral endothelial cells. Cells were exposed to normoglycemic condition in presence or not of each polyphenol-rich plant extract (10 µM GAE) or standard polyphenol (10 μM) for 24 h. In order to evaluate the cellular viability, the cells were counted by using the Trypan blue exclusion method (**A**). The mitochondrial metabolic activity was determined by the 3-(4,5-dimethyl-thiazol-2-yl)-2,5-diphenyl tetrazolium bromide (MTT) assay (**B**). Data are means ± SEM of three independent experiments.

In order to determine the possible effect of polyphenols on the cellular viability, bEnd3 cerebral endothelial cells were exposed to each polyphenol-rich plant extract or standard caffeic acid, chlorogenic acid, gallic acid, or quercetin. Cell counting data show that neither polyphenol-rich plant extracts nor standard polyphenols modulated the cellular viability (Figure 2A). Furthermore, any plant extract or standard polyphenol changed the mitochondrial metabolic activity of cerebral endothelial cells that were evaluated by MTT assay (Figure 2B), which suggested the absence of cytotoxic action on bEnd3 cerebral endothelial cells in our experimental conditions. 

### 3.3. Effect of Polyphenols on Intracellular ROS Levels on Cerebral Endothelial Cells in Hyperglycemic Condition

**Figure 3:**
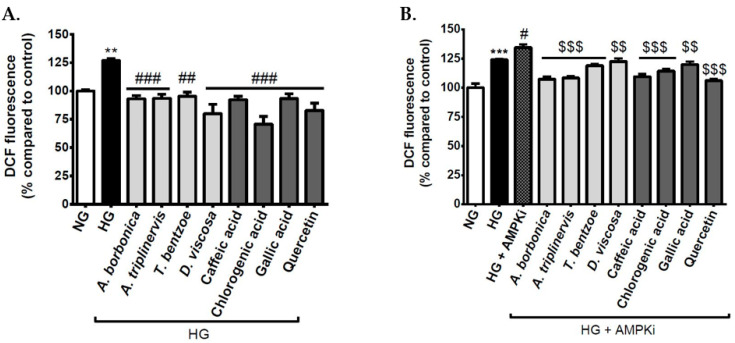
Effect of polyphenols on intracellular reactive oxygen species (ROS) levels on cerebral endothelial cells in hyperglycemic condition. Cells were exposed to normoglycemic condition (NG) or hyperglycemic condition (HG) in the presence or not of each polyphenol-rich plant extract (10 µM gallic acid equivalent (GAE)) or standard polyphenol (10 μM). Intracellular ROS levels were measured by the 2’,7’-dichlorodihydrofluorescein diacetate (DCFH-DA) assay (**A**). For the experiment with AMP-activated protein kinase (AMPK) pathway inhibitor, cells were pre-incubated with the Compound C (10 μM) during 1 h. Subsequently, the intracellular ROS levels were determined in the experimental conditions described above for normoglycemic (NG) and hyperglycemic (HG) conditions (**B**). Data are means ± SEM of three independent experiments. **: *p* < 0.01, ***: *p* < 0.005, as compared to NG. ##: *p* < 0.01 and ###: *p* < 0.005 as compared to HG. #: *p* < 0.05 as compared to HG. $$: *p* < 0.01 and $$$: *p* < 0.005 as compared to HG + AMPK pathway inhibitor.

During diabetes, oxidative stress plays a causal role in hyperglycemia-related neurovascular complications by inducing blood-brain barrier dysfunction. We determined the impact of an experimental hyperglycemia mimicked by high glucose concentration on ROS production from cerebral endothelial cells that were exposed or not to polyphenols. The data show that the exposure of cells to the hyperglycemic condition led to a significant increase in intracellular ROS levels reaching 126.91 ± 1.70% (Figure 3A). Noticeably, all polyphenol-rich plant extracts as well as standard caffeic acid, chlorogenic acid, gallic acid, and quercetin counteracted the deleterious effect of the hyperglycemic condition. These data suggest that hyperglycemic condition induced oxidative stress on cerebral endothelial cells and that polyphenols exerted a protective action. Such an antioxidant effect of polyphenol-rich plant extracts could be partly attributed to their free radical-scavenging and reducing capacities described above, and they may result from the presence of phytochemicals, like quercetin, caffeic, chlorogenic, and gallic acids in the medicinal plant extracts.

### 3.4. Effect of Signaling Pathway Inhibitors and Polyphenols on Intracellular ROS Levels on Cerebral Endothelial Cells in Hyperglycemic Condition

In order to better understand the molecular mechanisms that are involved in high glucose-induced ROS production from endothelial cells and the protective effect of polyphenols, bEnd3 cells were pre-incubated with specific inhibitors targeting NFκB or AMPK pathways, before ROS level measurement. The results show that the inhibition of NFκB pathway led to a decrease in ROS levels in both normoglycemic and hyperglycemic conditions, providing evidence for a causal role of NFκB in ROS production (Table 4). Conversely, the inhibition of AMPK pathway induced an elevation of ROS levels in both normoglycemic and hyperglycemic conditions, highlighting a protective role of AMPK against ROS production. Interestingly, the co-treatment of cells with AMPK pathway inhibitor and polyphenols during hyperglycemic condition abrogated the increase in ROS production exacerbated by AMPK pathway inhibitor (Figure 3B). This suggests that polyphenols may help to reduce redox damages that are related to AMPK pathway blockade and protect cerebral endothelial cells against high glucose-mediated oxidative stress. 

### 3.5. Effect of Polyphenols on SOD Activity on Cerebral Endothelial Cells in Hyperglycemic Condition

SOD activity was determined to elucidate the mechanisms involved in the ability of polyphenols to reduce oxidative stress mediated by hyperglycemic condition. The results show that the total SOD and Cu/ZnSOD activities were significantly elevated in cells exposed to hyperglycemic condition (Table 5). Interestingly, all of the polyphenol-rich plant extracts reversed the up-regulated activities of total and Cu/ZnSOD mediated by high glucose, except *D. viscosa* plant extract, which only modulated the activity of Cu/ZnSOD. Standard caffeic acid, chlorogenic acid, gallic acid, and quercetin also abrogated the hyperglycemic condition effect on total and Cu/ZnSOD activities. This suggests the possible involvement of these polyphenols in the bioactivity of medicinal plant extracts. Concerning MnSOD activity, it was unchanged in cells that were exposed to the hyperglycemic condition. Noteworthy, *A. triplinervis* plant extract, chlorogenic acid, gallic acid, and quercetin significantly increased MnSOD activity. Taken together, these findings demonstrate that Cu/ZnSOD was more particularly enhanced during high glucose-mediated oxidative stress. Such an enzymatic activation could result from the elevation of intracellular ROS levels induced by hyperglycemic condition that we reported above. Polyphenol-rich plant extracts may preserve the endogenous antioxidant defense system.

### 3.6. Effect of Polyphenols on the Expression of Genes Encoding Redox Markers on Endothelial Cells in Hyperglycemic Condition

**Figure 4:**
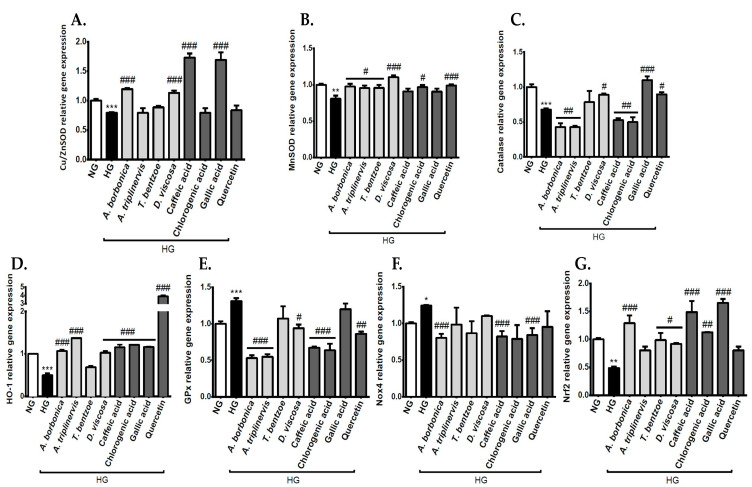
Effect of polyphenols on the expression of genes encoding redox enzymes and nuclear factor erythroid 2-related factor 2 (Nrf2) transcription factor on cerebral endothelial cells in hyperglycemic condition. Cells were exposed to normoglycemic condition (NG) or hyperglycemic condition (HG) in the presence or not of each polyphenol-rich plant extract (10 µM GAE) or standard polyphenol (10 μM). The expression of genes coding for Cu/ZnSOD (**A**), MnSOD (**B**), catalase (**C**), and heme oxygenase-1 (HO-1) (**D**), GPx (**E**), Nox4 (**F**), and Nrf-2 (**G**) was measured by RT-qPCR and normalized to GAPDH gene expression. The data are means ± SEM of three independent experiments. *: *p* < 0.05, **: *p* < 0.01 and ***: *p* < 0.005, as compared to NG. #: *p* < 0.05, ##: *p* < 0.01 and ###: *p* < 0.005 as compared to HG.

Redox status homeostasis is regulated by ROS-detoxifying enzymes, including Cu/ZnSOD, MnSOD, catalase, GPx, and HO-1, as well as ROS-producing enzymes, such as Nox4. It was relevant to assess the impact of polyphenol-rich plant extracts on the production of such redox enzymes. Results show on the one hand that the expression of genes coding for the antioxidant enzymes Cu/ZnSOD (Figure 4A), MnSOD (Figure 4B), catalase (Figure 4C), and HO-1 (Figure 4D) was down-regulated, and, on the other hand, that GPx gene expression (Figure 4E) was up-regulated in response to high glucose concentration. *A. borbonica* and *D. viscosa* plant extracts exerted a protective action against the modulation of Cu/ZnSOD, MnSOD, HO-1, and GPx gene expression mediated by hyperglycemic condition. *D. viscosa* plant extract also improved catalase gene expression. *A. triplinervis* plant extract protected against the alteration of MnSOD, HO-1, and GPx gene expression in high glucose-exposed cells. Regarding *T. bentzoe* plant extract, it counteracted MnSOD gene expression reduction mediated by hyperglycemic condition. Standard polyphenols were also able to counteract the impact of hyperglycemic condition on the expression of genes encoding redox enzymes, depending on the considered enzyme. Of note, all of the pure polyphenols abrogated the decrease in HO-1 gene expression caused by high glucose; and quercetin exerted a marked protective action by inducing a four-fold increase in HO-1 gene expression. Concerning the ROS-producing enzyme Nox4, its gene expression was up-regulated in hyperglycemic condition-exposed cells (Figure 4F). *A. borbonica* plant extract, caffeic, and gallic acids protected against Nox4 gene expression deregulation. It is well known that the transcription factor Nrf2 is involved in the enhancement of the expression of genes encoding antioxidant enzymes. Interestingly, high glucose concentration reduced by a two-fold factor Nrf2 gene expression (Figure 4G). This result is consistent with data described above showing the down-regulation of Cu/ZnSOD, MnSOD, catalase, and HO-1 gene expression in hyperglycemic condition. *A. borbonica*, *T. bentzoe*, and *D. viscosa* plant extracts as well as caffeic, chlorogenic, and gallic acids abrogated Nfr2 gene expression decrease during hyperglycemic condition. Collectively, these findings demonstrate that the hyperglycemic condition altered the cerebral endothelial cell redox status by promoting oxidative stress. Polyphenol-rich plant extracts exhibited protective antioxidant properties. Given that quercetin, caffeic, chlorogenic, and gallic acids exerted similar activities, these polyphenols could partly contribute to the biological effects of the medicinal plant extracts.

### 3.7. Effect of Polyphenols on Inflammatory Markers on Cerebral Endothelial Cells in Hyperglycemic Condition

**Figure 5:**
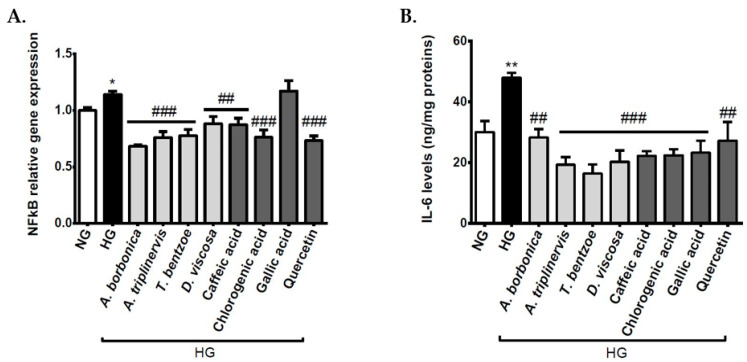
Effect of polyphenols on the production of inflammatory markers on cerebral endothelial cells in hyperglycemic condition. The cells were exposed to normoglycemic condition (NG) or hyperglycemic condition (HG) in the presence or not of each polyphenol-rich plant extract (10 µM GAE) or standard polyphenol (10 μM). The expression of NFκB gene was measured by RT-qPCR and normalized to GAPDH gene expression (**A**). The secretion of IL-6 was measured by ELISA kit (**B**). Data are means ± SEM of three independent experiments. *: *p* < 0.05 and **: *p* < 0.01 as compared to NG. ##: *p* < 0.01 and ###: *p* < 0.005 as compared to HG.

Hyperglycemia-mediated oxidative stress is known to cause the nuclear translocation of NFκB, which activates the transcription of several genes coding for pro-inflammatory cytokines, like IL-6. We evaluated the production of pro-inflammatory markers by measuring NFκB gene expression and IL-6 secretion. The data indicate that NFκB gene expression (Figure 5A) and IL-6 release (Figure 5B) were elevated in cells that were exposed to high glucose level, demonstrating a pro-inflammatory response of cerebral endothelial cells in hyperglycemic condition. Interestingly, all polyphenol-rich plant extracts and standard polyphenols exerted an anti-inflammatory action by lowering both NFκB gene expression and IL-6 secretion, except for gallic acid, without effect on NFκB gene expression.

### 3.8. Effect of Polyphenols on Vasoactive Markers on Cerebral Endothelial Cells in Hyperglycemic Condition

**Figure 6:**
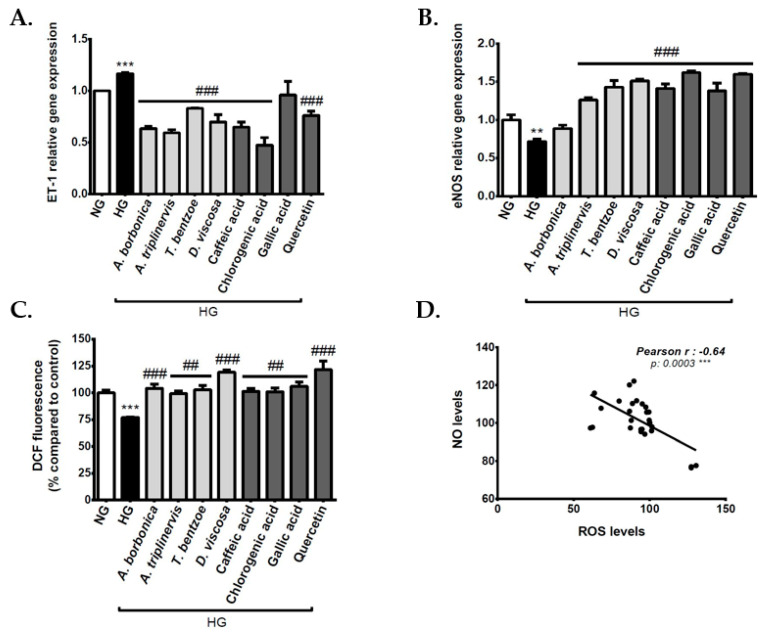
Evaluation of polyphenols on the production of vasoactive markers on cerebral endothelial cells in hyperglycemic condition. Cells were exposed to normoglycemic condition (NG) or hyperglycemic condition (HG) in the presence or not of each polyphenol-rich plant extract (10 µM GAE) or standard polyphenol (10 μM). The expression of genes coding for endothelin-1 (ET-1) (**A**) and endothelial nitric oxide synthase (eNOS) (**B**) was measured by RT-qPCR and normalized to GAPDH gene expression. Intracellular nitric oxide (NO) levels were measured by 4-amino-5-methylamino-2’,7’-difluorofluorescein (DAF-FM) assay (**C**). The statistical correlation between intracellular ROS and NO levels was established (**D**). Data are means ± SEM of three independent experiments. **: *p* < 0.01 and ***: *p* < 0.005 as compared to normoglycemic condition NG. ##: *p* < 0.01 and ###: *p* < 0.005 as compared to HG.

Endothelial cells are the major source of NO, which plays an important physiological role as a vasodilator factor. During hyperglycemia-mediated oxidative stress, it is established that ROS alter eNOS activity and NO production. Moreover, ROS may exacerbate the production of the vasoconstrictor factor ET-1. We evaluated the effect of polyphenols on these vasoactive markers. The results show that high glucose concentration caused an elevated expression of ET-1 gene expression (Figure 6A). Oppositely, hyperglycemic condition induced a down-regulation of eNOS gene expression (Figure 6B). Some polyphenol-rich plant extracts and standard polyphenols exerted a protective action against the deleterious effect of high glucose concentration on ET-1 and eNOS gene expression. Moreover, the hyperglycemic condition significantly decreased intracellular NO levels (Figure 6C), and all plant extracts counteracted this effect. Standard caffeic, chlorogenic and gallic acids, as well as quercetin exerted a similar protective action, suggesting their possible contribution to the plant extract benefits on NO generation during hyperglycemic condition. Noteworthy, there was a significant negative Pearson correlation between intracellular NO and ROS levels in cells that were exposed to the hyperglycemic condition (Figure 6D). This result suggests that oxidative stress caused by hyperglycemic condition could be associated with the dysfunction of cerebral endothelial cells through damaged production of NO.

### 3.9. Effect of Polyphenol Related Circulating Metabolites on Cerebral Endothelial Cell Viability, Redox, Inflammatory and Vasoactive Markers in Hyperglycemic Condition

**Figure 7:**
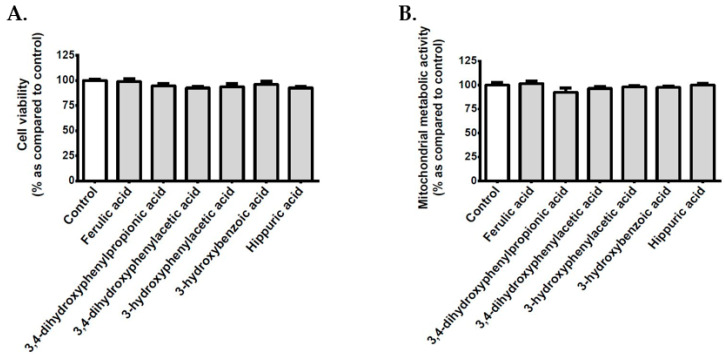
Effect of polyphenol related circulating metabolites on the viability and mitochondrial metabolic activity of cerebral endothelial cells. Cells were exposed to normoglycemic condition in presence or not of each compound (10 μM) for 24 h. The cellular viability was measured by counting cells through the Trypan blue exclusion method (**A**). The mitochondrial metabolic activity of cells was determined by the MTT assay (**B**). Data are means ± SEM of three independent experiments.

Given that polyphenols are poorly absorbed and extensively catabolized by the gut microflora, six related circulating metabolites were assessed for their ability to modulate cerebral endothelial cell viability as well as key redox, inflammatory, and vasoactive markers in hyperglycemic condition. These metabolites were ferulic, 3,4-dihydroxyphenylpropionic, 3,4-dihydroxyphenylacetic, 3-hydroxyphenylacetic, 3-hydroxybenzoic, and hippuric acids. Cell counting data (Figure 7A) and mitochondrial metabolic activity measurement (Figure 7B) show that polyphenol related circulating metabolites did not exert a cytotoxic effect on bEnd3 cerebral endothelial cells. Table 6 summarizes the data that were obtained regarding ROS production, NFκB transcriptional activity, IL-6 secretion, and NO levels in cells that were exposed to normoglycemic or hyperglycemic condition, in the presence or not of circulating metabolites. While the hyperglycemic condition significantly elevated intracellular ROS levels, only ferulic, 3,4-dihydroxyphenylpropionic, and 3,4-dihydroxyphenylacetic acids exerted an antioxidant effect by decreasing the ROS production. Of note, all of the polyphenol circulating metabolites exhibited anti-inflammatory properties by abrogating the elevation of NFκB activity and IL-6 release induced by high glucose condition. Furthermore, all of the polyphenol circulating metabolites protected against the decrease in intracellular NO vasodilator levels mediated by hyperglycemic condition. 

## 4. Discussion

During type 2 diabetes, hyperglycemia promotes oxidative stress, which induces cerebral endothelial dysfunctions and BBB alterations, aggravating cerebrovascular complications, such as stroke. The present study evaluated the protective effects of polyphenols on redox, inflammatory, and vasoactive markers of murine bEnd3 cerebral endothelial cells during hyperglycemic condition. This experimental hyperglycemic condition was mimicked by treating cells with a high glucose concentration (33 mM) after a glucose starvation related to normoglycemia (5.5 mM). Similar treatments with high glucose level were reported to mimic hyperglycemia in in vitro cellular models and led to demonstrate high glucose-caused redox and pro-inflammatory alterations that are associated with endothelial dysfunctions, such as altered permeability and reduced NO production [40,42,45,46]. Here, four characterized polyphenol-rich extracts from *A. borbonica*, *A. triplinervis*, *D. viscosa*, and *T. bentzoe* medicinal plants were assessed for their capacity to protect endothelial functions during hyperglycemic condition. Furthermore, quercetin, caffeic, chlorogenic, and gallic acids were used as reference polyphenols due to their identification in plant extracts. When considering that our previous study conducted on bEnd3 cerebral endothelial cells showed that hyperglycemic condition led to time-dependent redox alterations between 3–24 h of exposure [42], the same experimental condition was achieved here. Regarding the dose of 10 µM of polyphenols used, it is consistent with the pharmacological doses broadly used in literature and in our published studies [37,38,39,41,47]. This dose could be considered to be close to circulating concentrations reaching less than 10 µM in nutritional situations, given that polyphenols are poorly absorbed through the intestinal tract and that their bioavailability largely depends on their structure and microbial catabolism [27,32]. Of note, our present data demonstrate that polyphenols used at 10 μM did not alter the viability of bEnd3 cerebral endothelial cells after 24 h of treatment. Therefore, the biological effects of polyphenols observed could not be associated with a damaged cellular viability. Similar results were obtained when bovine aortic endothelial cells were treated with curcumin (10 μM) during 18 h of exposure. However, when the impact of different concentrations (0–30 μM) of curcumin was assessed, it was found that the cellular viability significantly decreased in the presence of 25 and 30 μM of curcumin [48]. Conversely, Chen et al. [49] demonstrated that a treatment with 10 and 25 μM of resveratrol for 24 h did not alter bEnd3 cell viability. These results suggest that the possible cytotoxic action of polyphenols might depend on their structure, the concentration used, the time of exposure, as well as the cell line considered. Several authors also reported polyphenol structure-antioxidant activity relationships [29]. Thus, it was essential here to characterize the chemical nature of polyphenols extracted from the medicinal plants selected. More particularly, leaves were used, since they constitute the most commonly and easily collected plant parts for the preparation of herbal remedies [50]. 

Our UPLC-ESI-MS-MS data show the presence of different types of polyphenols in medicinal plant extracts that are widely found in the human diet due to their abundance in several vegetables, fruits, and plant-derived beverages, such as coffee [27]. Indeed, for *A. borbonica* plant extract, the major polyphenols identified were caffeic and chlorogenic acids that are members of the family of phenolic acids well known to be abundant in many red fruits and coffee [27]. In agreement with our published data [38], quercetin and kaempferol glycosides that belong to the flavonoid family were also detected in *A. borbonica* plant extract. *D. viscosa* plant extract also contained different flavonoids, including the methylated form of quercetin, called isorhamnetin, procyanidins, and a trimethoxyflavone. While this trimethoxyflavone was reported as one of the bioactive phytochemicals responsible for the anti-inflammatory activity of *Artemisia asiatica* [51], quercetin, kaempferol, and procyanidins are flavonoids more commonly provided by the diet through the consumption of foods, such as onion, broccoli, grape, or cocoa. Noteworthy, literature data indicate that flavonoids may account for about two-thirds of the daily polyphenol intake in humans [27]. Quercetin glycosides and caffeic acid derivatives were also depicted in *A. triplinervis* plant extract. In addition, UPLC-ESI-MS-MS analysis helped to characterize the coumarin derivatives ayapin and ayapanin that we previously identified in *A. triplinervis* essential oil and correlated with oil composition variations, depending on the developmental stage and geographical location of the medicinal plant [52]. For *T. bentzoe* plant extract, the predominant polyphenols identified were gallic and ellagic acids as well as their sugar conjugates comprising the ellagitannins punicalin and punicalagin. Interestingly, these compounds may represent unique ellagitannins from specific dietary sources, like pomegranate, and be responsible for most of the antioxidant properties that were attributed to this fruit [53,54]. Altogether, our UPLC-ESI-MS-MS results demonstrate that the polyphenol composition was dependent on the medicinal plant considered. Concomitantly, the total polyphenol content ranging from 0.5 to 8.1 g GAE/100 g of plant also depended on the nature of the medicinal plant. It led to identifying *T. bentzoe* plant as the richest polyphenol source, followed by both *A. borbonica* and *D. viscosa* plants, and *A. triplinervis* plant. This agrees with literature data reporting that medicinal plants may naturally provide high levels of polyphenols similarly to dietary sources, such as tea, cocoa, grape, or curcuma [27,28,37,39]. Despite it being established that the total polyphenol content from fruits and vegetables varies depending on cultivars, climatic environment, cultural practices, and harvest conditions [55], there is still a lack of data regarding medicinal plants. Nevertheless, the total polyphenol content remains a relevant indicator of the antioxidant capacity of food matrix and a useful preliminary marker for identifying natural sources of polyphenols as functional foods [56]. 

An in vitro study was conducted on cerebral endothelial cells exposed to hyperglycemic condition in the presence or not of polyphenols to assess whether the presence of polyphenols in the medicinal plant extracts was associated with antioxidant activities. Our findings provide evidence for the antioxidant effects of medicinal plant polyphenols against high glucose-induced oxidative stress. Indeed, the results from DCFH-DA assay show that the hyperglycemic condition led to increased intracellular ROS levels. According to Kassab et al. [13], in hyperglycemic condition, glucose is phosphorylated and converted into fructose-6-phosphate, next into glyceraldehyde-3-phosphate and transformed into glycerol phosphate, a precursor of diacylglycerol which is a signaling molecule that is able to modulate protein kinase C activity involved in redox deregulation. Moreover, the overproduction of superoxide caused by hyperglycemia inhibits glucose-6-phosphate dehydrogenase that is the rate-limiting enzyme of the pentose phosphate pathway able to provide reducing equivalents to the endogenous antioxidant defense system [57]. Interestingly, medicinal plant polyphenols protected cerebral endothelial cells by decreasing ROS production elevated by hyperglycemic condition. Quercetin, caffeic, chlorogenic, and gallic acids also improved the ROS levels in hyperglycemic condition. This raises the possibility that such polyphenols could partly contribute to the antioxidant effects of plant extracts on cerebral endothelial cells. Accordingly, *A. borbonica* plant, quercetin, caffeic, chlorogenic, and gallic acids have previously been shown to decrease ROS levels in 3T3-L1 adipose cells that were exposed to metabolic and inflammatory stimuli, like hydrogen peroxide, TNFα, and bacterial lipopolysaccharides [38,39,41]. Here, cerebral endothelial cell pretreatment with specific inhibitors of intracellular signaling pathways highlights a causal role of NFκB, while a protective action of AMPK on redox changes. This is consistent with our published data demonstrating the causal involvement of NFκB, JNK, ERK, and PI3K pathways and the protective role of AMPK in ROS production of bEnd3 cells in hyperglycemic condition [42]. Moreover, we found that hyperglycemic condition induced a pro-inflammatory response of cerebral endothelial cells, by elevating NFκB gene expression and IL-6 release. Accordingly, several literature data provided evidence for the crosstalk between oxidative stress and inflammation, and showed that NFκB suppression limited redox damages in in vitro cell models as well as in diabetic rats [58,59,60]. Importantly, our present data show that polyphenol-rich plant extracts, as well as quercetin, caffeic, chlorogenic, and gallic acids exerted anti-inflammatory effects, by lowering NFκB and IL-6 production caused by high glucose on cerebral endothelial cells. In parallel, we found that all polyphenols tested abrogated the increase in ROS levels mediated by AMPK blockade in cells that were exposed to hyperglycemic condition. When considering that AMPK is a conserved key enzyme involved in cell energy homeostasis and able to counteract oxidative stress [61,62], polyphenols may help to reduce redox alterations related to AMPK suppression and, thus, to protect cerebral endothelial cells during hyperglycemia. Of note, Shen et al. [63] reported that polyphenols like quercetin up-regulate AMPK gene expression. This activation of AMPK prevents oxidative stress-induced vascular dysfunction via the increased phosphorylation and activation of eNOS. Jiang et al. [64] found that chlorogenic acid improved ROS and NO production by activating AMPK signaling pathway and eNOS activity on human aortic endothelial cells that were exposed to hypochlorous acid. 

The antioxidant properties of medicinal plant polyphenols on cerebral endothelial cells in hyperglycemic condition may result from their ability to scavenge and neutralize ROS, as demonstrated by DPPH assay. Indeed, through electronic delocalization on their phenolic nucleus, polyphenols can react with free radicals. Subsequently, the phenoxyl radicals produced are stabilized by the resonance effect of the phenolic nucleus. Moreover, the antioxidant activity of polyphenols depends on the number and the position of hydroxyl groups in the molecular structure [29]. Given that flavonoids are composed of three phenolic rings, such a chemical structure may explain why quercetin and the medicinal plant extracts containing quercetin derivatives exerted high free radical-scavenging and reducing capacities. The antioxidant properties of these molecules could also be associated with their capacity to modulate the mitochondrial function, keeping in mind that mitochondria constitute a main source of ROS [65]. Here, we show, for the first time, that all polyphenolic plant extracts, quercetin, and chlorogenic acid improved the mitochondrial MnSOD gene expression deregulated by hyperglycemic condition. In parallel, only *A. triplinervis* plant extract, quercetin, chlorogenic, and gallic acids significantly elevated MnSOD enzymatic activity, suggesting their possible specific impact on MnSOD mitochondrial marker. Our previous data demonstrated the ability of pure polyphenols, such as caffeic and gallic acids, to improve mitochondrial respiration and biogenesis in adipose cells that were exposed to hydrogen peroxide-mediated oxidative stress [66]. It will be relevant to evaluate the impact of medicinal plant extracts on mitochondrial functions of cerebral endothelial cells in hyperglycemic condition. It is known that cerebral endothelial cells are rich in mitochondria [67]. Additionally, brain is not well equipped with antioxidant defenses, despite its high consumption of oxygen [58], leading to an increased risk of brain damages during high glucose-induced oxidative stress. Thus, antioxidant strategies that aim to limit mitochondrial alterations at the cerebral level are of high interest. 

Oxidative stress occurs when there is an imbalance between the rate of ROS production and the capacity of the endogenous antioxidant defense to neutralize ROS. Our data indicate that plant polyphenols were able to regulate both ROS-producing and ROS-detoxifying systems, depending on the medicinal plant considered. One the one hand, only *A. borbonica* plant polyphenols as well as caffeic and gallic acids counteracted the up-regulation of the expression of the gene encoding the ROS-producing enzyme Nox4 in hyperglycemic condition. This agrees with our previous data showing the ability of *A. borbonica* plant polyphenols to abrogate Nox4 gene expression elevation in adipose cells that were exposed to oxidative stress induced by bacterial lipopolysaccharides [68]. A similar protective antioxidant effect of resveratrol was observed in bEnd3 cells through inhibition of high glucose-induced Nox activation and ROS generation [49]. Nox4 is the most abundant vascular Nox isoform, located in the mitochondria. Once activated, Nox4 leads to an increased production of ROS that triggers the nuclear translocation of NFκB, leading to pro-inflammatory cytokine production [16]. A persistent elevation of Nox4 mRNA levels was described in human aortic endothelial cells that were exposed for 24–72 h to hyperglycemic condition. This event was associated with the nuclear translocation of NFκB and its binding to Nox4 gene promoter [69]. Concordantly, our present study demonstrates NFκB involvement in redox changes mediated by high glucose in bEnd3 cells. In vivo studies reported increased levels of Nox4 protein in cerebral endothelial cells from mouse ischemic brains and stroke patient samples. A link between the level of Nox4 protein, brain infarct volume, and neurological improvements was depicted in Nox4^−/−^ mice [70]. Here, our data highlighting the capacity of polyphenols to target both Nox4 and NFκB suggest the relevance to assess their possible benefits in in vivo animal models exposed to cerebrovascular complications, such as stroke. On the other hand, medicinal plant polyphenols also improved major antioxidant factors, including SOD, catalase, GPx, HO-1, and Nrf2. More precisely, we found that Cu/ZnSOD activity was up-regulated in hyperglycemic condition, whereas MnSOD activity was not modulated by high glucose. Accordingly, Weidig et al. [71] observed a significant elevation of Cu/ZnSOD activity in coronary endothelial cells that were exposed to 22 mM of glucose. This may be explained by the fact that cytosolic Cu/ZnSOD is recognized as the first line of the endogenous defense system to neutralize superoxide radicals [72]. Medicinal plant polyphenols regulated SOD activity on cerebral endothelial cells in hyperglycemic condition, which suggested their ability to preserve the endogenous antioxidant defense system. Moreover, the hyperglycemic condition down-regulated the expression of Cu/ZnSOD, MnSOD, catalase, and HO-1 genes and up-regulated GPx gene expression, while polyphenols counteracted these effects. Accordingly, *A. borbonica* plant polyphenols were previously shown to up-regulate SOD gene expression on adipose cells that were exposed to oxidative stress [39]. It was also reported that quercetin, detected in *A. borbonica*, *A. triplinervis* and *D. viscosa* plants, improved HO-1 gene expression on endothelial cells exposed to hyperglycemic condition. Of note, Li et al. [73] demonstrated that quercetin significantly elevated HO-1 gene expression when human aortic endothelial cells were exposed to the pro-inflammatory mediators lipopolysaccharides, by regulating the Nrf2 signaling pathway. Additionally, quercetin was shown to regulate SOD enzymatic activity in a traumatic brain injury model via Nrf2 signaling pathway [74]. Nrf2 redox-sensitive transcriptional factor is recognized to play a crucial role in cellular defense mechanisms against a variety of stresses. During oxidative stress condition, Nrf2 is translocated into the nucleus and binds to the antioxidant response element of target genes, such as SOD, catalase, GPx, and HO-1, to regulate their transcription rate, helping to restore redox homeostasis [75]. Recently, we showed that high glucose-induced oxidative stress was associated with altered Nrf2 gene expression and nuclear translocation in cerebral endothelial cells [42]. The present study demonstrates that all medicinal plant polyphenols, except *A. triplinervis* plant, abrogated the down-regulation of Nrf2 gene expression mediated by hyperglycemic condition in cerebral endothelial cells. Quercetin, caffeic, chlorogenic, and gallic acids exerted a similar protective action, raising the possibility for their contribution to the beneficial effect of medicinal plant extracts. Consistently, literature data reported that polyphenols, such quercetin and caffeic acid, regulate Nrf2 production [76,77]. According to Nabavi et al. [78], Nrf2 may provide a new therapeutic target for the treatment of diabetic microvascular complications. Authors also emphasized the relevance of antioxidant strategies while using polyphenols to target Nrf2. In Nrf2^−/−^ rat and diabetic mouse models, Nrf2 activation led to an improvement of vascular function and decreased vasoconstriction [18,79]. Such a link between oxidative stress and endothelial dysfunctions was found in the present study, through the evidence of a negative correlation between intracellular ROS levels and NO vasodilator production. Interestingly, our data demonstrate that all medicinal plant extracts counteracted the decrease in NO production induced by the hyperglycemic condition. Except for *A. borbonica* plant, all polyphenol-rich plant extract also protected against the down-regulation of eNOS gene expression mediated by high glucose. Conversely, medicinal plant polyphenols suppressed the up-regulation of ET-1 vasoconstrictor gene expression that was caused by high glucose. These data demonstrate the protective action of medicinal plant extracts against the altered production of vasoactive markers in hyperglycemic condition and suggest their capacity to ameliorate vasodilation and attenuate vasoconstriction. Similar beneficial effects were detected for quercetin, caffeic, and chlorogenic acids, emphasizing their possible involvement in the bioactivity of plant extracts. Accordingly, other studies showed that polyphenols, like quercetin and chlorogenic acid, protected endothelial cells from hypochloride-caused oxidative stress and improved ex vivo aortic vessel function by enhancing eNOS activity and NO release [63,64]. Taken together, our findings demonstrate for the first time that the French medicinal plants *A. borbonica*, *A. triplinervis*, *D. viscosa*, and *T. bentzoe* constitute natural sources of antioxidant and anti-inflammatory polyphenols that are able to improve vasoactive markers, and could be used as innovative therapeutic strategies against cerebrovascular disorders that are associated with hyperglycemia. The novel comparative analysis we performed regarding the medicinal plant polyphenolic composition helps to identify three medicinal plants with high levels of polyphenols, namely *T. bentzoe*, *A. borbonica*, and *D. viscosa*, as compared to *A. triplinervis* plant, which contains a lower polyphenol content. Concomitantly, the comparison of the biological activities of plant extracts on the same cellular model and in similar experimental conditions highlights the absence of cytotoxic action for all plant extracts tested. This underlines the possibility of their use in preclinical studies, in order to identify the most bioactive extract for future investigations in humans. On the one hand, all of the polyphenolic plant extracts were able to modulate redox, inflammatory, and vasoactive markers in cerebral endothelial cells, with a degree of efficiency depending on the medicinal plant considered. This provides new perspectives on the advantage of the use of a mixture of natural polyphenolic compounds when compared to the use of pure polyphenols for nutritional strategies. Such mixtures of natural polyphenols may also lead to their synergistic action through the impact of plant matrix on polyphenol chemical stability and cell accessibility. Our previous study contributed to show that a crude curcuminoid-rich extract from *Curcuma longa* exerts stronger antioxidant and anti-inflammatory effects on adipose cells than curcumin-enriched fractions [37]. On the other hand, the use of pure compounds helps to hypothesize their contribution to the mechanism of action of plant extracts, and to emphasize their relevance as therapeutic potentials for pharmacological strategies against cerebrovascular complications related to hyperglycemia. 

Limitations of this study warrant mention. Polyphenol bioavailability is known to be low, given that, depending on their chemical structure, polyphenols are poorly absorbed through the intestinal tract and extensively catabolized by the gut microflora into microbial metabolites. Previously, we contributed to identifying the parent polyphenols quercetin, caffeic, chlorogenic, and gallic acids in urine samples that were collected from humans after the intake of polyphenol-rich beverages, demonstrating their ability to be absorbed in in vivo condition [80]. In parallel, literature data [32,33] and our previous studies [81,82,83] led to detect ferulic, 3,4-dihydroxyphenylpropionic, 3,4-dihydroxyphenylacetic, 3-hydroxyphenylacetic, 3-hydroxybenzoic and hippuric acids as circulating metabolites in rats and humans after consumption of polyphenols like caffeic, chlorogenic and gallic acids, quercetin, and procyanidins. When considering the presence of such polyphenols on the medicinal plant extracts we used here, it was relevant to assess the effect of their related circulating metabolites on cerebral endothelial cells. Importantly, our data show, for the first time, that the polyphenol circulating metabolites tested improve ROS production, NFκB transcriptional activity, IL-6 secretion, and NO levels in cerebral endothelial cells during the hyperglycemic condition. This result emphasizes their possible involvement in the capacity of medicinal plant polyphenols to improve redox, inflammatory, and vasoactive markers in in vivo condition. It will be of interest to determine the molecular mechanism implicated in the bioactivity of polyphenol circulating metabolites. Another limitation of the present study is that polyphenol uptake mechanism by cells remains poorly understood. Literature data indicate that the uptake of polyphenols, such as flavonoids, depends on cellular types. Rather than different levels of passive diffusion, this may be explained by a greater level of intracellular metabolism and faster rate of export from different cells [84,85]. It is also assumed that polyphenol transport through the BBB is limited and associated with the inhibition of efflux transporters, such as P-glycoprotein [86,87]. Absorbed phenolic acids, like caffeic acid, were found to accumulate in the brain at nanomolar concentrations [88] and exert neuroprotective effects during cerebral ischemic injury [89]. Our recent study conducted on a mouse stroke model co-exposed to hyperglycemia and *A. borbonica* plant polyphenols or pure caffeic acid, led to demonstrate the presence of caffeic acid and its circulating metabolite ferulic acid in the infarcted hemisphere. Moreover, we found that the capacity of polyphenols to decrease cerebral infarct volume and hemorrhagic transformation aggravated by hyperglycemia was associated with an improvement of the production of vascular endothelial-cadherin used as a BBB integrity marker, and with a reduction of neuroinflammation [40]. Further work will be needed in order to assess the gut absorption, metabolism, and ability of other medicinal plant polyphenols to target cerebrovascular cells in in vivo diabetic and ischemic conditions. Given that UPLC-ESI-MS-MS analysis identified polyphenols such as quercetin, caffeic, chlorogenic and gallic acids in medicinal plant extracts, the biological effects of standard molecules were assessed. It will be relevant to evaluate their role as active principles after fractionation and purification from medicinal plants. However, it is not excluded that other phytochemicals detected like kaempferol, trimethoxyflavone, ellagic acid or ellagitannins may be involved. Literature data provided evidence for their antioxidant and anti-inflammatory properties [38,51,53,54]. In our study, polyphenol-rich plant extracts were prepared through a pharmacological approach based on aqueous acetonic extraction. The most traditionally employed method for preparation of herbal remedies is infusion, which consists of soaking the plant part in very hot water [50]. Recently, *A. borbonica* plant infusion was shown to exert antioxidant properties on in vitro and in vivo models exposed to glycoxidative condition [90]. It will be of interest to evaluate the benefits of infusions that were prepared from the medicinal plants tested here, during cerebrovascular complications related to diabetes.

## 5. Conclusions

**Figure 8:**
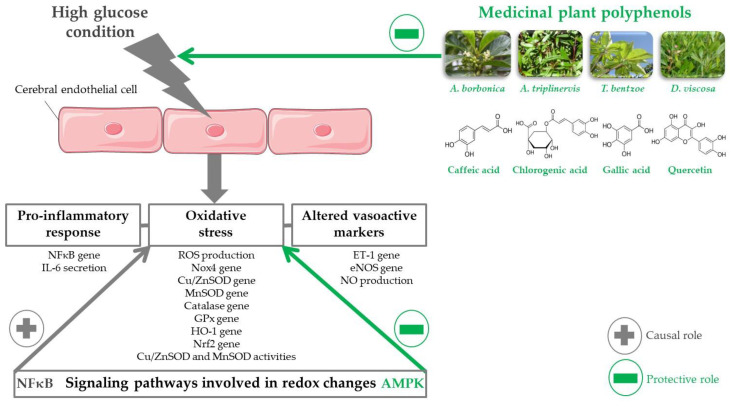
Overview of the molecular targets involved in the protective effects of medicinal plant polyphenols on cerebral endothelial cells in hyperglycemic condition. High glucose condition increased ROS levels and deregulated key redox factors comprising Nox4, SOD, catalase, GPx, HO-1, and Nrf2. Medicinal plant polyphenols attenuated oxidative stress mediated by hyperglycemic condition. Cell preconditioning with inhibitors of signaling pathways showed the causal role of NFκB while a protective action of AMPK on redox changes. Hyperglycemic condition promoted a pro-inflammatory status by elevating NFκB gene expression and IL-6 secretion, and damaged ET-1, eNOS and NO vasoactive marker production. Interestingly, medicinal plant polyphenols protected against the deleterious effects of hyperglycemic condition. Quercetin, caffeic, chlorogenic and gallic acids identified as predominant plant polyphenols exerted similar protective effects.

This study demonstrates that medicinal plant polyphenols attenuated oxidative stress and improved inflammatory and vasoactive markers on cerebral endothelial cells in hyperglycemic condition. Figure 8 summarizes the molecular targets that are involved in polyphenol action. Whereas the hyperglycemic condition induced oxidative stress by deregulating several redox factors, including ROS, Nox4, SOD, catalase, GPx, HO-1, and Nrf2, medicinal plant polyphenols limited oxidative stress mediated by hyperglycemic condition. Cell pretreatment with inhibitors of signaling pathways led to show the causal role of NFκB, while a protective action of AMPK on redox changes. Hyperglycemic condition induced a pro-inflammatory response by increasing NFκB gene expression and IL-6 secretion. Moreover, it altered ET-1, eNOS, and NO vasoactive marker production. Importantly, medicinal plant polyphenols protected against the deleterious effects of hyperglycemic condition. Quercetin, caffeic, chlorogenic, and gallic acids identified as predominant plant polyphenols, and related circulating metabolites exerted similar beneficial activities. A better understanding of the molecular mechanisms that are involved in polyphenol uptake by cerebral endothelial cells is needed. Preclinical studies on diabetic animal models will also help to assess the ability of polyphenols to target cells in in vivo condition and protect against cerebrovascular complications during type 2 diabetes.

## Figures and Tables

**Table 1:** Primers used for reverse-transcribed-quantitative polymerase chain reaction (RT-qPCR) analysis.

Mouse Gene	Sequence
Catalase	Forward CCT-CCT-CGT-TCA-GGA-TGT-GGT-T Reverse CGA-GGG-TCA-CGA-ACT-GTG-TCA-G
Cu/Zn Superoxide dismutase (Cu/ZnSOD)	Forward GCA-GGG-AAC-CAT-CCA-CTT Reverse TAC-AAC-CTC-TGG-ACC-CGT
endothelial Nitric oxide synthase (eNOS)	Forward CTG-TGG-TCT-GGT-GCT-GGT-C Reverse TGG-GGC-AAC-TTG-AAG-AGT-GTG
Endothelin-1 (ET-1)	Forward CCC-TTT-GCA-GAA-TGG-ATT-AT Reverse CTG-TAG-TCA-ATG-TGC-TCG-GT
Glyceraldehyde-3-phosphate dehydrogenase (GAPDH)	Forward TTC-ACC-ACC-ATG-GAG-AAG-GC Reverse GGC-ATG-GAC-TGT-GGT-CAT-GA
Glutathione peroxidase (GPx)	Forward TGC-TCA-TTG-AGA-ATG-TCG-CGT-CTC Reverse AGG-CAT-TCC-GCA-GGA-AGG-TAA-AGA
Heme oxygenase-1 (HO-1)	Forward GGT-CAT-GGC-TTC-CTT-GTA-C Reverse AGT-GAG-GCC-CAT-ACC-AGA-AG
MnSOD	Forward ATG-TTG-TGT-CGG-GCG-GCG Reverse AGG-TAG-TAA-GCG-TGC-TCC-CAC-ACG
Nuclear factor kappa B (NFκB)	Forward GTG-ATG-GGC-CTT-CAC-ACA-CA Reverse CAT-TTG-AAC-ACT-GCT-TTG-ACT
NADPH oxidase 4 (Nox4)	Forward GAT-CAC-AGA-AGG-TCC-CTA-GCA-G Reverse GTT-GAG-GGC-ATT-CAC-CAA-GT
Nuclear factor erythroid 2-related factor 2 (Nrf2)	Forward TTG-GCA-GAG-ACA-TTC-CCA-T Reverse GCT-GCC-ACC-GTC-ACT-GGG

**Table 2:** Compounds identified in polyphenol-rich extracts from medicinal plants.

Medicinal Plant	Retention Time (min)	Molecular Weight (Da)	Parent Ions	Fragment Ions	Identified Compound
*A. borbonica*	2.5	354	353	191	Chlorogenic acid isomer
	3.5	354	353	191	Chlorogenic acid
	3.7	354	353	191, 173	Chlorogenic acid isomer
	5.7	610	609	301	Quercetin-hexoside-ramnoside
	6.0	594	593	447, 285	Kaempferol-hexoside-ramnoside
	6.2	594	593	455, 285	Kaempferol-hexoside-ramnoside
	6.4	516	515	353, 191	Dicaffeoylquinic acid
	6.6	516	515	353, 191	Dicaffeoylquinic acid
	6.9	516	515	353, 191	Dicaffeoylquinic acid
*A. triplinervis*	3.2	154	153	109	Protocatechuic acid
	4.4	180	179	/	Caffeic acid
	5.0	356 610	355 609	193 301, 179	Feruoyl-hexoside Quercetin-hexoside-ramnoside
	5.2	464	463	301	Quercetin-hexoside
	5.5	550	549	505, 463, 301	Quercetin-hexoside-malonate
	6.1	696	695	533, 515, 371, 209	Tricaffeoyl-glucarate
	6.3	194	193	/	Isoferulic acid
	6.8	190	191 *	163, 133 *	Ayapin
	7.4	176	177 *	145, 133, 121 *	Ayapanin (Herniarin)
*T. bentzoe*	1.2	482	481	301	Hexahydroxydiphenoyl (HHDP)-hexoside
	1.4	482	481	301	HHDP-hexoside
	2.3	170	169	125	Gallic acid
	2.7	782	781	721, 601, 299, 271	Punicalin
	3.0	934	933	/	Galloyl-punicalin
	3.2	934	933	915, 781, 721, 601	Galloyl-punicalin
	3.4	470	469	425	Valoneic acid dilactone
	3.5	1084	1083	781, 601	Punicalagin
	3.8	1084	1083	781, 601	Punicalagin
	3.9	186	1085	783, 601, 451	Digalloyl-gallagyl-hexoside
	4.0	634	633	463, 301	HHDP-galloyl-hexoside
	4.1	1086	1085	933, 721, 601	Digalloyl-punicalin
	4.3	786	785	633, 483	HHDP-digalloyl-hexoside
	4.9	938	937	785, 767, 741, 465, 301	Tri-galloyl-HHDP-glucose
	5.2	302	301	/	Ellagic acid
	5.3	940	939	787, 769, 617	Pentagalloyl-hexoside
*D. viscosa*	3.8	1152	1151	981, 863, 711	Procyanidin tetramer type A
	4.2	578	557	451, 425	Procyanidin dimer B2
	4.6	864	863	711, 693, 559	Procyanidin trimer type A
	5.4	624	623	315	Isorhamnetin-hexoside-rhamnoside
	9.0	344	343	329, 301, 283	5,7-Dihydroxy-3,6,4′-trimethoxyflavone (Santin)

Polyphenol-rich extracts from medicinal plants were analyzed by UPLC-ESI-MS-MS at 280 nm. The phytochemicals were detected according to their retention time and the *m*/*z* ratio of their parent ions [M − H]^−^ and fragments at negative mode, except for the parent ions [M + H]^+^ and fragments that were detected at positive mode and indicated by *.

**Table 3:** Total polyphenol contents and free radical-scavenging and reducing activities of plant extracts.

	Total Polyphenol Contents (g GAE/100 g)	DPPH Reduced (%)
Plants		
*A. borbonica*	3.44 ± 0.11 ^b,c^	89.50 ± 1.24 ^e^
*A. triplinervis*	0.58 ± 0.02 ^a,c,d^	67.99 ± 1.58 ^f^
*T. bentzoe*	8.14 ± 0.27 ^a,b,d^	92.43 ± 0.42
*D. viscosa*	3.22 ± 0.12 ^b,c^	89.95 ± 1.41
Standard antioxidants		
Vitamin C	-	93.78 ± 0.65
Caffeic acid	-	92.58 ± 0.63
Chlorogenic acid	-	89.36 ± 0.75 ^e^
Gallic acid	-	94.04 ± 0.06
Quercetin	-	92.56 ± 0.54

Total polyphenol contents from medicinal plant extracts were determined by Folin-Ciocalteu assay and expressed as g gallic acid equivalent (GAE)/100 g plant. Free radical-scavenging and reducing capacities of plant polyphenols (100 µM GAE), vitamin C, quercetin, caffeic, chlorogenic and gallic acids (100 µM) were measured by DPPH assay and expressed as % DPPH reduced. Data are means ± SEM of three independent experiments. a: *p* < 0.005 as compared to *A. borbonica*, b: *p* < 0.005 as compared to *A. triplinervis*, c: *p* < 0.005 as compared to *T. bentzoe*, d: *p* < 0.005 as compared to *D. viscosa*, e: *p* < 0.05 and f: *p* < 0.005 as compared to vitamin C positive control.

**Table 4:** Effect of nuclear factor kappa B (NFκB) and AMP-activated protein kinase (AMPK) inhibition on intracellular ROS levels of cerebral endothelial cells during hyperglycemic condition.

Cell Conditions	Intracellular ROS Levels (% of Control)		
	Vehicle	NFκB Inhibition	AMPK Inhibition
Normoglycemic condition	100 ± 2.12	80.38 ± 2.92 *	137.89 ± 3.19 ***
Hyperglycemic condition	127.75 ± 2.66 ***	80.28 ± 3.21 ###	142.16 ± 2.88 ##

Cells were preconditioned with NFκB and AMPK inhibitors (10 µM), for 1 h before the exposition to DCFH-DA probe for 45 min. Subsequently, the cells were co-exposed to the inhibitors (10 µM) and 5.5 mM or 33 mM of glucose to mimic normoglycemic and hyperglycemic conditions, respectively. Intracellular ROS levels were measured by DCFH-DA assay. The data are means ± SEM of three independent experiments. *: *p* < 0.05 and ***: *p* < 0.005 as compared to normoglycemic condition. ##: *p* < 0.01 and ###: *p* < 0.005 as compared to hyperglycemic condition.

**Table 5:** Effect of polyphenols on superoxide dismutase (SOD) activity on cerebral endothelial cells in Hyperglycemic condition.

Cell Conditions	Total SOD Activity (U/g Protein)	Cu/ZnSOD Activity (U/g Protein)	MnSOD Activity (U/g Protein)
NG	9.45 ± 0.41	2.28 ± 0.13	6.09 ± 0.13
HG	13.28 ± 0.76 ***	10.77 ± 0.54 ***	4.68 ± 0.54
HG + *A. borbonica*	10.85 ± 0.36 #	6.27 ± 0.25 ##	5.10 ± 0.54
HG + *A. triplinervis*	9.09 ± 0.37 ###	4.05 ± 0.93 ###	9.01 ± 1.24 ##
HG + *T. bentzoe*	9.69 ± 0.31 ###	7.14 ± 0.09 #	4.84 ± 1.37
HG + *D. viscosa*	13.08 ± 0.14	7.41 ± 0.77 #	5.08 ± 0.77
HG + Caffeic acid	8.73 ± 0.37 ###	3.80 ± 0.36 ###	6.42 ± 0.71
HG + Chlorogenic acid	7.47 ± 0.43 ###	3.24 ± 0.04 ###	8.30 ± 0.04 #
HG + Gallic acid	11.16 ± 0.40 #	1.22 ± 0.64 ###	11.33 ± 0.64 ###
HG + Quercetin	8.90 ± 0.73 ###	3.65 ± 1.50 ###	10.42 ± 1.86 #

Cells were exposed to normoglycemic condition (NG) or hyperglycemic condition (HG) in presence or not of each polyphenol-rich plant extract (10 μM GAE) or standard polyphenol (10 μM). Subsequently, total SOD, Cu/ZnSOD, and MnSOD activities were measured. Data are means ± SEM of three independent experiments. ***: *p* < 0.005 as compared to NG. #: *p* < 0.05, ##: *p* < 0.01 and ###: *p* < 0.005 as compared to HG.

**Table 6:** Effect of polyphenol related circulating metabolites on redox, inflammatory, and vasoactive markers on cerebral endothelial cells in hyperglycemic condition.

Cell Conditions	ROS Levels (%)	NFκB/SEAP Activity (%)	IL-6 Secretion (ng/mg Proteins)	NO Levels (%)
NG	100.00 ± 2.46	100.00 ± 1.11	30.01 ± 3.64	100.00 ± 2.54
HG	118.75 ± 1.89 ***	133.69 ± 10.46 **	47.95 ± 1.61 **	70.51 ± 0.94 ***
HG + Ferulic acid	84.33 ± 4.51 ###	104.40 ± 0.23 ##	31.96 ± 2.09 #	80.08 ± 1.13 ##
HG + 3,4-Dihydroxyphenylpropionic acid	104.16 ± 3.95 #	100.29 ± 3.41 ##	26.62 ± 8.30 #	83.42 ± 1.66 ###
HG + 3,4-Dihydroxyphenylacetic acid	84.17 ± 4.14 ###	102.03 ± 1.10 ##	23.50 ± 7.90 #	80.62 ± 0.30 ###
HG + 3-Hydroxyphenylacetic acid	109.38 ± 1.16	104.31 ± 2.46 ##	19.34 ± 1.80 ###	83.65 ± 1.36 ###
HG + 3-Hydroxybenzoic acid	107.21 ± 0.74	102.89 ± 0.58 ##	28.13 ± 7.20 #	79.77 ± 0.93 #
HG + Hippuric acid	113.72 ± 0.94	95.78 ± 3.18 ##	26.69 ± 3.00 ##	91.53 ± 3.36 ###

Cells were exposed to normoglycemic condition (NG) or hyperglycemic condition (HG) in the presence or not of each polyphenol related circulating metabolite (10 μM). Intracellular ROS levels was measured by DCFH-DA assay. NFκB/SEAP activity and IL-6 secretion were determined by Quanti-Blue assay and ELISA kit, respectively. The intracellular NO levels were measured by DAF-FM assay. Data are means ± SEM of three independent experiments (three cellular passages), except for NFκB/SEAP activity and IL-6 secretion measurement, which were performed twice (two cellular passages). **: *p* < 0.01 and ***: *p* < 0.005 as compared to NG. #: *p* < 0.05, ##: *p* < 0.01 and ###: *p* < 0.005 as compared to HG.
